# An unusual case of small bowel volvulus due to pneumatosis cystoides intestinalis: Case report and literature review

**DOI:** 10.1016/j.ijscr.2024.109328

**Published:** 2024-02-01

**Authors:** Carlos Eduardo Rey Chaves, Juan Fernando Fonseca, Natalia Ballen, Andrés Bravo, Laura Becerra Sarmiento, Fania Gabriela Parra Blanco, Laura Felisa Peña Carvajalino, Maria Camila Azula Uribe

**Affiliations:** a Estudiante de posgrado Cirugía General, Pontificia Universidad Javeriana, Facultad de Medicina. Bogotá, Colombia; b Médica General, Pontificia Universidad Javeriana, Facultad de Medicina, Bogotá, Colombia; c Estudiante de posgrado Patología, Pontificia Universidad Javeriana, Facultad de Medicina, Bogotá, Colombia; d Patologa, Pontificia Universidad Javeriana, Facultad de Medicina, Hospital Universitario San Ignacio, Bogotá, Colombia; e Cirugía General, Pontificia Universidad Javeriana, Facultad de Medicina, Hospital Universitario San Ignacio, Bogotá, Colombia

**Keywords:** Pneumatosis cystoides intestinalis, Intestinal ischemia, Surgery, Case report

## Abstract

**Introduction and importance:**

Pneumatosis cystoides intestinalis (PCI) is an uncommon condition characterized by intramural gas accumulation in the intestinal submucosa. Idiopathic or secondary is presented with non-specific clinical signs; in some cases, diagnosis is incidental. Its acute presentation is uncommon, and surgical management could be performed in selected cases.

**Case presentation:**

We present the case of an 85-year-old woman with 3 days of abdominal pain, 6 months of weight loss, and abdominal distension after meals. Abdominal computed tomography evidenced PCI at the small intestine with changes due to intestinal ischemia and internal mesenteric hernia. Intestinal resection and lateral-lateral mechanical anastomosis were performed with no complications after 90 days of follow-up.

**Clinical discussion:**

PCI is an infrequent and benign condition; pathophysiology is, to date, poorly understood. Idiopathic or secondary to other gastrointestinal pathologies are described. The final diagnosis is performed with histopathological analysis. Nevertheless, in some cases, the benign nature could be presented as an acute abdomen, and surgical management should be in the physician's armamentarium.

**Conclusion:**

PCI is an uncommon and benign entity. Nevertheless, in some cases, it could be presented as an acute abdomen. The surgical approach is appropriate, safe, and feasible.

## Highlights

• Small bowel obstruction due to neumatosis cystoides is a rare clinical entity.
• Vascular occlusion in cases of pneumatosis cystoids could develop chronic mesenteric ischemia.
• There is no gold standard treatment for this condition, and management should be individualized.

## 1 Introduction and importance

Pneumatosis cystoides intestinalis (PCI) results as a rare gastrointestinal complication characterized by intramural accumulation of gas within thin-walled cysts or bullae in the colon's intestinal submucosa and the small intestine's subserosa [[1], [2], [3], [4], [5]]. Located predominantly in the transverse, descending and sigmoid colon [1]. A higher proportion of this condition is presented in men, with a ratio of 2.4:1 [1].

Etiologically, it can be divided into primary or idiopathic (15 %) and secondary or associated with other diseases, which occurs in 85 % of cases [1]. PCI presents as nonspecific gastrointestinal symptoms or asymptomatic presentation and can be an incidental finding in radiological studies and colonoscopies [1]. CT has helped increase the recognition of this pathology, which can be misdiagnosed for intestinal necrosis or perforation [5]. To determine the approach, it is crucial to correlate clinical presentation with laboratory and imaging tests to evaluate whether a medical approach or emergency surgical intervention is appropriate [5].

## 2 Case presentation

**Fig. 1:**
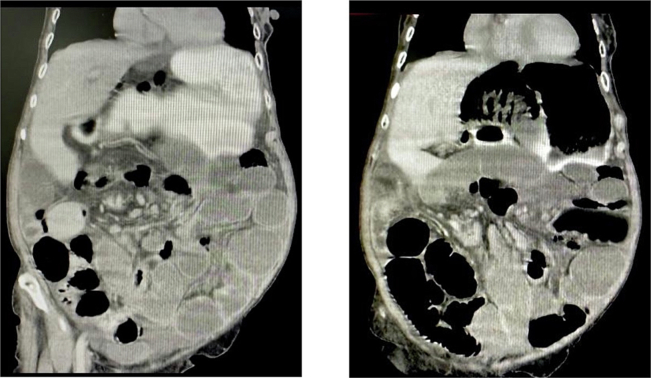
Abdominal CT: Trans-mesenteric internal hernia associated with internal obstruction and intestinal ischemia.

**Fig. 2:**
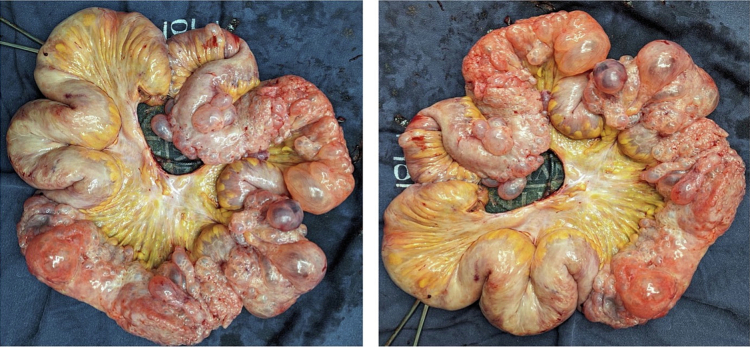
Extensive ileum pneumatosis cystoides with focal changes of necrosis and mesenteric thrombosis.

**Fig. 3:**
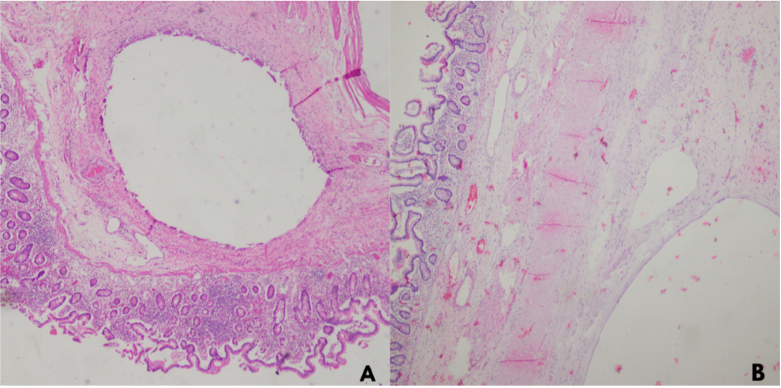
Empty pseudocyst in a muscular layer adjacent to the giant cell layer (HE 4x). B) Pseudolipomatosis, associated with distortion of the villi (HE 4x).

After ethical and institutional approval and previous informed consent were filled, following SCARE guidelines [6]. We present a case of an 85-year-old woman with no comorbidities who attended the emergency service on account of a 3-day clinical presentation of diffuse abdominal pain associated with distention, nausea, and vomiting. The patient refers to a 6-month evolution time of abdominal discomfort after meals, nausea, and weight loss (7 kg in 3 months). At physical examination, tachycardia (100 beats per minute), abdominal distention, and generalized pain were found without signs of peritoneal irritation. Serum analysis was requested, normal white blood cell count, hemoglobin values, renal function, and C reactive protein (WBC 10.400, Neutrophils 89 %, hemoglobin 14 g/dL, creatinine 0.9; and CRP 1.2). Blood arterial gas analysis was requested and evidenced metabolic acidosis and no hyperlactatemia (pH 7.3, PCO2 36 MMHG, PO2 64.2 MMHG HCO3 19 MMOL/L, Lactate 1.2). A plain abdominal x-ray was requested with pneumoperitoneum associated with small bowel dilatation and indirect signs of intestinal pneumatosis. Due to clinical stability and no signs of peritoneal irritation, abdominal contrast computed tomography was performed, with evidence of trans mesenteric internal hernia associated with intestinal obstruction and intestinal ischemia (Fig. 1). Due to this radiological evidence, surgical management was decided. Median laparotomy was performed, with evidence of extensive pneumatosis cystoides evidenced between 310 and 430 cm from the Treitz angle, in association with mesenteric volvulus from the ileum with chronic changes. There is no evidence of intestinal perforation (Fig. 2). Patients did not require vasopressors; the normal temperature was evidenced; intestinal resection and later-lateral anastomosis with mechanical sutures using the Barcelona technique were performed, and primary fascial closure was reached. Nil per oz. was indicated for the first 48 h, and progressive restoration of oral intake was indicated with adequate tolerance; no intensive care unit was required, and the in-hospital length of stay was 6 days. Postoperative diarrhea was evidenced in the first 30 days of surgery. Histological analysis of the surgical piece demonstrates arterial thrombosis with focal changes due to ischemia and pneumatosis cystoid intestinal with necrotic change (Fig. 3). No postoperative complications were evidenced after 60 days of follow-up.

## 3 Discussion

Pneumatosis cystoides intestinalis consists of the presence of gaseous cysts containing nitrogen, hydrogen, and carbon dioxide within the intestinal submucosa and serosa [[1], [2], [3]]. Cysts are distributed predominantly in the large intestine in (46 %) of cases (especially sigmoid), small intestine (27 %), small intestine and colon (7 %), and stomach (5 %) [1,2]. It is rare to encounter this condition in the duodenum and rectum [1]. PCI is divided into primary (15 %) and secondary (85 %) forms, which occur secondary to diseases such as digestive tract stenosis, obstructive pulmonary disease, abdominal surgery, abdominal injury, and malnutrition [2].

Three theories have been proposed about the etiology of the disease. The bacterial theory behind etiopathogenesis suggests it results from excess hydrogen gas secondary to intraluminal bacterial fermentation and altered partial nitrogen pressure within the intestinal wall [1,5]. The other proposition, the mechanical theory, suggests this condition results in the formation of gas cysts through the layer of the bowel wall due to high luminal pressure from intestinal obstruction [1,3,5]. Finally, the pulmonary theory suggests that chronic lung diseases such as chronic obstructive pulmonary disease, asthma, and intestinal pneumonia conduct to alveolar rupture, leading to mediastinal emphysema and gas release along the aorta and mesenteric blood vessels leading into the intestinal wall [2].

The pathophysiological mechanisms attributed to the generation of the disease include inflammation, physical damage of intestinal mucosa, nutritional imbalance, dysbiosis, altered gastrointestinal motility, and immune system dysfunction [1]. Post-surgery, chemotherapy, Acarbose, Trichloroethylene (TCE), scleroderma, connective tissue disease, and pulmonary illness are some of the predisposing factors that have been associated with PCI [1,4]. Complications of systemic sclerosis and systemic lupus erythematosus are related to abnormal immune function regulation and prolonged corticoid use, which results in atrophy of intestinal mucosa that promotes the formation of cysts in the intestinal submucosa [4]. Molecular targeted agents such as epidermal growth factor receptor (EGFR) and anti-vascular endothelial growth factor (VEGF) likely potentially increase intestinal toxicity related to PCI [4]. In our case, there is no evidence of other comorbidity related to the diagnosis, and we suspect an idiopathic condition of our patient, with a chronic illness related to the large compromise of the small intestine.

Asymptomatic or non-specific gastrointestinal symptoms such as abdominal distention, diarrhea (53 %), constipation, hematochezia (12 %), rectal tenesmus, nausea and vomiting (14 %), abdominal pain (59 %), and weight loss, are some of the ambiguous clinical manifestations, that lack specificity and inflict a challenge for physicians in the diagnosis of PCI [1,2,4]. Diagnosis incorporates radiography, CT, colonoscopy and abdominal ultrasound [2]. Abdominal CT confirms the diagnosis with the presence of cysts in a honeycomb or bunch of grapes pattern in the intestinal wall [1]. Severe forms include the presence of intestinal dilatation, arterial or venous occlusion, ascites, and porto-mesenteric or porto-hepatic venous gas [1]. In our case, the patient presented with a chronic intestinal obstruction with an acute presentation due to ileal mesenteric volvulus presented as an acute abdomen.

In general, the existence of free gas in the abdominal cavity corresponds to the perforation of hollow viscera [8,9]. Pneumoperitoneum is the presence of gas in the peritoneal cavity, represented by subdiaphragmatic radiolucency, which suggests rupture of hollow viscera in about 85–95 % of cases [[8], [9], [10]]. This radiological sign, as well as a clinical presentation of abdominal distension, febrile syndrome, absence of leukocytosis, and peritoneal signs, puts pneumatosis cystoid intestinalis as a diagnostic possibility [[8], [9], [10], [11], [12]]. Nevertheless, in patients with PCI, pneumoperitoneum could reflect the rupture of one or more cysts without visceral rupture, as in our case, intraoperatively, we don't evidence any sign of hollow visceral perforation; nevertheless, there are changes in the small intestine and in the mesentery that reflects chronic changes.

It is essential to be able to detect abnormalities in normal intestinal gas distribution and identify abnormal collections to distinguish the presence of an alteration. According to the clinical severity, it is classified into two groups, potentially lethal intestinal pneumatosis, where the rupture of cysts causes pneumoperitoneum, pneumoretroperitoneum, and air in the omentum in <3 % of cases, and benign intestinal pneumatosis [1]. It is important to rule out other non-surgical etiologies of pneumoperitoneum which in asymptomatic patients can resolve spontaneously and not require unnecessary surgeries [[8], [9], [10], [11], [12], [13]].

Pneumatosis cystoides consist of a benign condition, which generally resolves with bowel rest, antibiotics (metronidazole, tinidazole, rifaximin, quinolones), and supportive care [1,[5], [6], [7], [8], [9]]. An important part of treatment relies on inhalational oxygen therapy, as an elevated venous oxygen concentration from high-flow oxygen therapy is believed to decrease the partial pressure of nitrogenous gases, attenuating gaseous cysts, releasing gases contained within the cysts then being refiled with oxygen which is then metabolized leading to resolution and eradicating anaerobic gut bacteria due to the toxic properties of oxygen on this bacteria [3,5]. Some studies suggest the optimal therapy is with hyperbaric oxygen, and to decrease the recurrence rate, it should be continued until two days after the disappearance of cysts [2,3]. Exceptions to the usual course of the disease include approximately 3 % of patients who develop complications such as pneumoperitoneum, intestinal volvulus, obstruction, hemorrhage, and intestinal ischemia [2,[5], [6], [7], [8], [9]]. The presence of symptoms such as abdominal pain and peritoneal irritation, accompanied by intestinal obstruction, bleeding, porto-mesenteric venous gas, decreased arterial pH, an elevated lactic acid or amylase level, suggest possible pneumoperitoneum or bowel infarction and require immediate surgical intervention due to the risk of developing ischemic colitis and necrosis [1,[5], [6], [7], [8], [9]]. Primary operative indications include: obstructive symptoms, white blood count >12 c/mm^3 or portal vein gas in CT findings especially when the patient is older than 60 years old because of the high mortality rate of PCI [[8], [9], [10], [11], [12], [13]]. Secondary indications to perform surgery are the presence of sepsis or signs of acidosis [2]. In our case, tomographic findings that suggest an internal hernia with intestinal ischemia suggest a potentially fatal condition, and for that reason, a surgical approach was preferred. Intraoperatively intestinal resection with mechanical anastomosis was performed, considering clinical condition and absence of vasopressors during surgery. Histopathological analysis confirms arterial thrombosis with ischemic and necrotic focal changes at the mesentery. After 30 days of follow-up the patient presented with diarrhea, possibly related with intestinal adaptation after resection. With a follow-up of 90 days, there is no evidence of postoperative complications.

## 4 Conclusion

Pneumatosis cystoides intestinalis is an uncommon entity, frequently sub-diagnosed. Even Though it's a benign disease, it could be presented as an acute abdomen. This is an uncommon case regarding this presentation with chronic intestinal obstruction with acute presentation of intestinal ischemia due to small intestine mesenteric volvulus. Surgical management, when appropriate, is a safe and feasible approach.

## Provenance and peer review

Not commissioned, externally peer-reviewed.

## Ethical approval

Following approval of our Institutional Review Board and ethical committee, all procedures performed in studies involving human participants were in accordance with the ethical standards of the institutional and/or national research committee, and with the 1964 Helsinki Declaration and its later amendments or comparable ethical standards. Informed consent was obtained from all individual participants included in the study.

## Funding

This research did not receive any specific grant from funding agencies in the public, commercial, or not-for-profit sectors.

## Author contribution

C.E.R.C and J.F·F had the research idea.

C.E.R.C, J.F.F., N·B, A.B and L.B·S, Participated in drafting the article and revised it critically for important intellectual content.

C.E.R.C, J.F.F., N·B, A.B, F.G.P.·B, L.F.P·C and M.C.A.U made substantial contributions to the conception and design, acquisition of data, analysis, and interpretation of data.

## Guarantor

Carlos Eduardo Rey Chaves.

## Research registration number

N/A.

## Conflict of interest statement

None of the authors declare any conflict of interest.

## Data Availability

The datasets used and/or analyzed during the current study are available from the corresponding author upon reasonable request.
